# The spectrum of cardiac disease in the West Region of Cameroon: a hospital-based cross-sectional study

**DOI:** 10.1186/1755-7682-6-44

**Published:** 2013-10-20

**Authors:** Ahmadou M Jingi, Jean Jacques N Noubiap, Philippe Kamdem, Edvine Wawo Yonta, Elvis Temfack, Charles Kouam Kouam, Samuel Kingue

**Affiliations:** 1 Department of Internal Medicine and Specialties, Faculty of Medicine and Biomedical Sciences, University of Yaoundé I, Yaoundé, Cameroon; 2 Internal Medicine Unit, Edéa Regional Hospital, PO Box 100, Edéa, Cameroon; 3 Centre Médical de la Trinité, Bafoussam, Cameroon; 4 Department of Internal Medicine, Yaoundé Teaching Hospital, Yaoundé, Cameroon; 5 Department of Internal Medicine, Douala General Hospital, Douala, Cameroon; 6 Department of Internal Medicine, Bafoussam Regional Hospital, Bafoussam, Cameroon; 7 Department of Internal Medicine, Yaoundé General Hospital, Yaoundé, Cameroon

**Keywords:** Cardiac disease, Sub-Saharan Africa, Cameroon

## Abstract

**Background:**

Cardiovascular disease is a growing public health problem in Africa. The extent of heart disease in Cameroon remains largely unknown. This study aimed at reporting the etiology of cardiac disease in a cardiologic clinic situated in a semi-urban area in the West region of Cameroon.

**Methods:**

This is an analysis of echocardiographic diagnosis of cardiac disease done between July 2008 and October 2010 at the “Centre Medical de la Trinité” in the West region of Cameroon. Data included age, sex and echocardiographic findings.

**Results:**

A total of 1252 patients presented with abnormal echocardiograms, 60.4% (n = 756) being female and 85.8% (n = 1074) aged over 50 years. Overall, the most important conditions were hypertensive heart disease (41.5%, n = 520) and cardiomyopathies (30.5%, n = 382). Among patients aged less than 10 years, congenital heart diseases were the most frequent (52.4%, n = 22), and rheumatic heart disease was the most important cardiac condition in patients aged 10 to 19 years (62.1%, n = 18) and those aged 20 to 39 years (53.3%, n = 8). Congenital heart diseases included persistent ductus arteriosus (27.6%, n = 8), tetralogy of Fallot (20.7%, n = 6) and inter-atria/interventricular communication (20.7%, n = 6).

**Conclusion:**

Hypertension is the leading cause of cardiac disease among the elderly in our setting, emphasizing the necessity to strengthen the preventive strategies against hypertension in Cameroon. Rheumatic heart disease and congenital heart disease frequent in children and youths highlight the need of early detection and treatment of throat infections, and of routine cardiac surgery services in Cameroon.

## Introduction

Sub-Saharan Africa (SSA) is currently going through an epidemiologic transition characterized by the coexistence of both acute infections and chronic diseases which are becoming increasingly important [1]. Surveys of the global burden of disease indicate that non-communicable diseases will become the leading cause of mortality worldwide in 2030, with the majority of burden occurring in developing countries [2]. Following Acquired immunodeficiency syndrome (AIDS), cardiovascular diseases are today the second most common cause of death in SSA, and the leading among people aged over 30 years [3,4].

There is a scarcity of data on the causes of heart failure in SSA. Previous studies have reported non-ischemic cardiopathies to be the most frequent cause of heart failure; with rheumatic heart disease, hypertensive heart disease and cardiomyopathy accounting for over 75% of cases in most series [3,5-10].

The prevalence and pattern of congenital and acquired cardiac diseases vary both within and between regions and countries [11-13]. These variations are due to difference in genetic background and environmental factors such as dietary habit, physical activity and the burden of infectious diseases. In Cameroon, hypertensive heart disease, cardiomyopathies and valvular heart diseases have been found to account respectively for 54.5%, 26.3% and 24.5% of causes of adult chronic heart failure in an urban setting [14]. However, data on occurrence and pattern of cardiac disease in Cameroon are scarce, especially among populations from rural and semi-rural areas. In this paper we report on the etiology of cardiac disease in a cardiologic outpatient clinic in a semi-urban setting of the West region of Cameroon. Data will contribute to design effective intervention for prevention, diagnosis and treatment of cardiac disease in Cameroon and other sub-Saharan African countries.

## Methods

The study was conducted in the “Centre Medical de la Trinité”, one of the two cardiologic clinics in the West Region of Cameroon which has a population estimated at 1,800,000 inhabitants [15]. This region is characterized by a very limited access to effective interventions for prevention, diagnosis and treatment of cardiovascular diseases. Patients included in this study were referred from all health facilities of the region to the study clinic for investigation and/or treatment after the clinical suspicion or diagnosis of a cardiac disease. Between July 2008 and October 2010, age, sex and cardiac diagnosis of every patient with an abnormal echocardiogram presenting to the clinic were recorded. This study is a retrospective analysis of these recorded data.

Trans-thoracic bi-dimensional guided M-mode echocardiography using commercially available echocardiography equipment (HP Sonos 2000 Color Doppler ver. A.2, HP Color) and a 4–7 Megahertz transducer was performed according to the American College of Cardiology and the American Heart Association [16]. Echocardiographic examination was performed in the parasternal long axis, short axis, apical four chamber and occasionally in the subcostal and suprasternal views. Indices analyzed included the left ventricle telesystolic diameter (LVIDS), left ventricle telediastolic diameter (LVIDD) and the ejection fraction (EF).

Hypertensive heart disease was diagnosed in the presence of any or combination of the following abnormalities: left ventricular systolic dysfunction (ejection fraction < 50%), left ventricular hypertrophy (indexed LV mass > 51 g/m2.7), and dilated left atrium, a surrogate of impaired LV filling (left atrial diameter > 3.8 cm in women and > 4.2 cm in men).

Valvular heart diseases were documented based on the following: (i) Mitral stenosis: presence of thickened and calcified mitral valve leaflets, loss of the classic M-shaped pattern of a normal mitral valve, diastolic dooming and restriction of the mitral valve leaflet motions. (ii) Mitral Regurgitation: poor coaptation of the mitral valve leaflets in systole, thickened leaflets, dilated and hyperdynamic left ventricle. (iii) Aortic stenosis: presence of calcified aortic valve, reduction in aortic cusp separation, highly echo reflectant aortic valve leaflets. (iv) Aortic regurgitation: poor coaptation of the aortic cusps in diastole, dilated left ventricles and fine fluttering of the anterior mitral valve in diastole [17]. Post rheumatic valvulopathy was defined by the presence of any definite evidence of mitral or aortic valve regurgitation seen in two planes by the TTE, accompanied by at least two of the following three morphologic abnormalities of the regurgitating valve: restricted leaflet mobility, focal or generalized valvular thickening, and abnormal sub-valvular thickening [17]. Dilated cardiomyopathy was diagnosed when there was dilated heart chambers with normal or decreased wall chambers as well as impaired LV systolic function [18]. Pericardial effusion was diagnosed when there is echo free space between the visceral and parietal pericardium. The echocardiography was performed by an experienced cardiologist.

The collected data were coded, entered and analyzed using STATA version 8.2. Descriptive analysis was made to describe the spectrum of disease.

Prior to commencing the study, hospital local institutional ethical clearance was obtained.

## Results

A total of 1252 patients presented with abnormal echocardiograms during the study period. There were 756 (60.4%) female and 496 (39.6%) males. About 86% of patients were aged over 50 years. Overall, the most important conditions were hypertensive heart disease (41.5%) and cardiomyopathies (30.5%), Table 1. Among patients aged less than 10 years, congenital heart diseases were the most frequent (52.4%), and rheumatic heart disease was the most important cardiac condition in patients aged 10 to 19 years (62.1%) and those aged 20 to 39 years (53.3%). Congenital heart diseases included persistent ductus arteriosus (27.6%), tetralogy of Fallot (20.7%) and inter-atria/interventricular communication (20.7%). Accounting for 78.5%, dilated cardiomyopathies were the most frequent myocardiopathies (Table 2).

**Table 1:** Echocardiographic diagnosis among the 1252 patients

**Heart diseases**	**Frequency (%)**					**Total**
	**0-9 years**	**10-19 years**	**20-39 years**	**40-49 years**	**≥50 years**	
Congenital heart disease	22 (52.4)	3 (10.3)	4 (26.7)	0 (0)	0 (0)	29 (2.3)
Rheumatic heart disease	12 (28.6)	18 (62.1)	8 (53.3)	2 (2.2)	2 (0.2)	42 (3.4)
Hypertensive heart disease	0 (0)	0 (0)	0 (0)	8 (8.7)	512 (47.7)	520 (41.5)
Cardiomyopathies	0 (0)	2 (6.9)	3 (20)	49 (53.2)	328 (30.5)	382 (30.5)
Endocarditis	6 (14.3)	4 (13.8)	0 (0)	8 (8.7)	3 (0.3)	21 (1.7)
Ischaemic heart disease	0 (0)	0 (0)	0 (0)	0 (0)	30 (2.8)	30 (2.4)
Pericarditis	0 (0)	2 (6.9)	0 (0)	25 (27.2)	61 (5.7)	88 (7)
Others	2 (4.7)	0 (0)	0 (0)	0 (0)	138 (12.8)	140 (11.2)
**Total**	42 (3.4)	29 (2.3)	15 (1.2)	92 (7.3)	1074 (85.8)	1252 (100)

Proportions of heart diseases are reported according to each age group.

**Table 2:** Some specific heart diseases

**Specific heart diseases**		**Frequency (%)**
**Congenital heart diseases (N = 29)**	Persistent ductus arteriosus	8 (27.6)
	Tetralogy of Fallot	6 (20.7)
	Inter-atria/interventricular communication	6 (20.7)
	Ebsteins disease	2 (6.9)
	Left ventricular atresia	2 (6.9)
	Others	5 (17.2)
**Cardiomyopathies (N = 382)**	Dilated cardiomyopathy	300 (78.5)
	Hypertrophic cardiomyopathy	72 (18.9)
	Restrictive cardiomyopathy	10 (2.6)

## Discussion

This study aimed at determining the spectrum of heart disease in a semi-urban setting of Cameroon. The leading cardiac disease in this study was hypertensive heart disease (41.5%). It is well established that hypertension forms the foundation of cardiovascular diseases in Africa [4,19]. Kingue et al. reported that hypertension accounted for 54.49% of causes of adult chronic heart failure in Yaoundé, Cameroon [14]. These findings point out the burden of hypertension in Cameroon. In facts, hypertension in Cameroon remains underdiagnosed and undertreated leading to hypertensive heart disease and chronic heart failure. In other studies conducted in SSA, hypertension has become the 1^st^ or the 2^nd^ leading cause of heart disease [6-10,20-22]. This increasing burden of hypertension is a major pattern of the epidemiologic transition facing by SSA countries characterized by more people living in urban areas with risky dietary habit and physical inactivity [1]. The burden of hypertension in SSA has been evaluated by Kearney et al., indicating a higher absolute number of people living with hypertension in economically developing countries, including SSA [23]. According to these authors, in 2000 the estimated total number of people with hypertension was 639 million (625–654 million) in economically developing countries, a number which is projected to increase by 80% in 2025 and reach 1.15 billion [23]. These alarming data highlight the crucial need of aggressive preventive policies against hypertension in SSA. Our findings also show that hypertensive heart disease was highly frequent only in patients aged over 50 years. This suggests that there was probably a significant gap time between the onset of hypertension and his consequence on heart function in our patients. Therefore, early diagnosis of hypertension and treatment should significantly delay the occurrence of hypertensive heart disease in our setting.

As also reported by Kingue et al. myocardiopathies were the second most frequent cause of heart disease in this study [14]. These findings collaborate with those of others studies done in SSA which shown a significant contribution of myocardiopathies [6-10,20-22]. Dilated cardiomyopathies were the most frequent cardiomyopathies, accounting for 78.5% of cases in our study. This predominance was found elsewhere [9,10,22].

Other studies have reported a significant contribution of rheumatic heart disease in SSA settings [9-14,22]. On the contrary, rheumatic heart disease accounted for only 3.4% of all cases. This could be explained by the fact that rheumatic heart disease occurs in a relative young population, and people aged less than 40 years represented only 6.9% of our study population. Much more, we found that rheumatic heart disease was the leading condition in the groups of patients aged 10 to 19 years (62.1%) and 20–39 years (53.3%). This suggests that in our setting rheumatic heart disease occurs frequently in youths, but because of the lack of cardiac surgical treatment, the affected patients may die approximately before the age of 40 years. This early mortality of patients affected by rheumatic heart disease is supported by the paucity of cases seen in patients aged over 40 years. Rheumatic heart disease results from repeated acute rheumatic fever attacks following exposure to Group A streptococci (GAS) throat infections [12]. Our findings therefore point out the need of early detection and treatment of throat infections in our setting.

We also found that endocarditis was more frequent in young patients. This could be related to congenital and rheumatic heart disease frequent in this age group and which favor microbial colonization of the altered endocardial anatomy. Pericarditis which was found to be relatively frequent in patients aged between 40 to 49 years could be linked to HIV/AIDS.

There is only one cardiac surgery center for more than 20 million people living in Cameroon [22,24]. About half of cases in patients aged less than 10 years were congenital heart diseases. This emphasizes the need of scaling up cardiac surgery services in Cameroon.

Our study is limited by the fact that the data reported are only based on echocardiography. The contribution of arrhythmias is therefore lacking. Details on the description and severity of the cardiac diseases reported are also lacking.

## Conclusion

Our study shows that hypertension is the leading cause of cardiac disease in our setting, and especially among the oldest, emphasizing the necessity to improve the preventive strategies against hypertension and other cardiovascular risk factors in Cameroon. Rheumatic heart disease and congenital heart disease frequent in children and youths highlight the need of early detection and treatment of throat infections, and of routine cardiac surgery services in Cameroon.

## Competing interests

The authors declare that they have no competing interests.

They have not benefited from any sponsorship and funding.

## Authors’ contributions

AMJ designed the study, collected and analyzed the data, and drafted the manuscript. JJNN contributed in study design, data analysis and drafted the manuscript. PK contributed to study design, performed all echocardiography examinations and collected the data. EWY, EK, CKK and SK contributed in study design and critically reviewed and revised the manuscript. All authors approved the final version of the manuscript.

## References

[B1] WHO The global burden of disease: 2004 update 2004 Geneva: WHO

[B2] Mathers C Loncar D Projections of global mortality and burden of disease from 2002 to 2030 PLoS Med 2006 6 e442 10.1371/journal.pmed.0030442 17132052 PMC1664601

[B3] Gaziano T Global burden of cardiovascular disease Braunwald's Heart Disease: A Textbook of Cardiovascular Medicine 2007 8 Philadelphia: Elsevier Saunders 1 21

[B4] Gaziano T Cardiovascular disease in the developing world and its cost-effective management Circulation 2005 6 23 3547 3553 10.1161/CIRCULATIONAHA.105.591792 16330695

[B5] Lopez D Assessing the burden of mortality from cardiovascular disease World Health Stat 1993 6 91 96 8303909

[B6] Damasceno A Dzudie A Mayosi B Heart failure in sub-Saharan Africa: time of action J Am coll cardiol 2007 6 1688 1693 10.1016/j.jacc.2007.07.030 17950152

[B7] Damasceno A Mayosi BM Sani M Ogah OS Mondo C Ojji D The causes, treatment, and outcome of acute heart failure in 1006 Africans from 9 countries Arch Intern Med 2012 6 18 1386 94 10.1001/archinternmed.2012.3310 22945249

[B8] Hunt SA Abraham WT Chin MH Feldman AM Francis GS Ganiats TG ACC/AHA 2005 Guideline Update for the Diagnosis and Management of Chronic Heart Failure in the Adult: a report of the American College of Cardiology/American Heart Association Task Force on Practice Guidelines (Writing Committee to Update the 2001 Guidelines for the Evaluation and Management of Heart Failure): developed in collaboration with the American College of Chest Physicians and the International Society for Heart and Lung Transplantation: endorsed by the Heart Rhythm Society J Am Coll Cardiol 2005 6 12 e154 235

[B9] Habte B Alemseqed F Tesfaye D The pattern of cardiac diseases at the cardiac clinic of Jimma University specialised hospital, south West Ethiopia Ethiop J Health Sci 2010 6 2 99 105 22434967 10.4314/ejhs.v20i2.69435 PMC3275840

[B10] Ogah OS Adegbite GD Akinyemi RO Adenisa JO Alabi AA Udofia OI Spectrum of heart diseases in a new cardiac service in Nigeria: an echocardiographic study of 1441 subjects in Abeokuta BMC Res Notes 2008 6 98 10.1186/1756-0500-1-98 18957102 PMC2585576

[B11] Brickner ME Hillis LD Lange RA Congenital heart disease in adults: 1 N Engl J Med 2000 6 256 263 10.1056/NEJM200001273420407 10648769

[B12] Zuhlke L Mirabel M Marijon E Congenital heart disease and rheumatic heart disease in Africa: recent advances and current priorities Heart 2013 6 21 1554 1561 10.1136/heartjnl-2013-303896 23680886 PMC3812860

[B13] Mbewu A Mbanya JC Jamison A Cardiovascular disease Disease and Mortality in Sub-Saharan Africa 2006 2 Washington (DC): World Bank

[B14] Kingue S Dzudie A Menanga A Akono M Ouankou M Muna W A new look at adult chronic heart failure in Africa in the age of the Doppler echocardiography: experience of the medicine department at Yaounde General Hospital Ann Cardiol Angeiol (Paris) 2005 6 5 276 83 10.1016/j.ancard.2005.04.014 16237918

[B15] Bureau central des recensements et des études de population du Cameroun Rapport de présentation des résultats définitifs de 3ème recesement général de la population humaine Accessed on http://www.statistics-cameroon.org/downloads/Rapport_de_presentation_3_RGPH.pdf

[B16] Cheitlin MD Alpert JS Armstrong WF Aurigemma GP Beller GA Bierman FZ Davidson TW Davis JL Douglas PS Gillam LD ACC/AHA Guidelines for the Clinical Application of Echocardiography. A report of the American College of Cardiology/American Heart Association Task Force on Practice Guidelines (Committee on Clinical Application of Echocardiography). Developed in Collabobration with the American Society of Echocardiography Circulation 1997 6 6 1686 1744 10.1161/01.CIR.95.6.1686 9118558

[B17] Prendergast BD Banning AP Hall RJ Valvular heart disease: recommendations for investigation and management - Summary of guidelines produced by a working group of the British Cardiac Society and the Research Unit of the Royal College of Physicians J R Coll Physicians Lond 1996 6 4 309 15 8875375 PMC5401593

[B18] Cardiomyopathies Report of a WHO expert committee World Health Organ Tech Rep Ser 1984 6 7 64 6428049

[B19] Kengne AP Awah KP Fezeu L Mbanya JC The burden of high blood pressure and related risk factors in urban and sub-Saharan Africa: evidences from Douala in Cameroon Afr Health Sci 2007 6 38 44 17604525 10.5555/afhs.2007.7.1.38 PMC2366125

[B20] Ntusi NB Mayosi BM Epidemiology of heart failure in Sub-Saharan Africa Expert Rev Cardiovasc Ther 2009 6 2 169 80 10.1586/14779072.7.2.169 19210213

[B21] Thiam M Cardiac insufficiency in the African milieu Bull Soc Pathol Exot 2003 6 217 218 14582299

[B22] Tantchou Tchoumi JC Ambassa JC Kingue S Giamberti A Cirri S Frigiola A Butera G Occurrence, aetiology and challenges in the management of congestive heart failure in sub-Saharan Africa: experience of the cardiac centre in Shishong Cameroon Pan Afr Med J 2011 6 11 22121420 10.4314/pamj.v8i1.71059 PMC3201578

[B23] Kearney PM Whelton M Reynolds K Muntner P Whelton PK He J Global burden of hypertension: analysis of worldwide data Lancet 2005 6 9455 217 223 15652604 10.1016/S0140-6736(05)17741-1

[B24] Budzee A Tantchou Tchoumi JC Ambassa JC Gimberti A Cirri S Fridiola A Buetra G The Cardiac Center of Shisong Hospital, the first cardio-surgical center in West and Central Africa is inaugurated Pan Afr Med J 2010 6 4 21119989 PMC2984304

